# Supplementary material for: Molecular Mechanisms of Phenylpropane-Synthesis-Related Genes Regulating the Shoot Blight Resistance of Bambusa pervariabilis × Dendrocalamopsis grandis
Source: Int J Mol Sci. 2022 Jun 17;23(12):6760. doi: 10.3390/ijms23126760 (PMC9224335; doi:10.3390/ijms23126760)
Supplement: Supplementary file 1 [file ijms-23-06760-s001.zip › ijms-1752432-supplementary.pdf]

Supplementary Materials

Table S1. Primers used for PCR.

Name	Primer sequence
Zf-COF	TCGAAATCGATAAGCTTGGATCCAGCACCCATGGAACCTGATG
Zr-COF	ATCTCATTAAAGCAGGACTCTAGAAGAGCGTGTTGTCGTAGCC
Ff-COF	AAATAATTATTTCCTTACCAATTGGAGCACCCATGGAACCTGATG
Fr-COF	CGCTCGAGGAATTCGGTACCAGAGCGTGTTGTCGTAGCC
Zf-CAD	TCGAAACTGATAAGCTTGGATCCTGCGGGATCTGCCACACTGACA
Zr-CAD	ATCTCATTAAAGCAGGACTCTAGAAGTACTGCTCGACGTTGGCCTT
Ff-CAD	AAATAATTATTTCCTTACCAATTGGTGCGGGATCTGCCACACTGACA
Fr-CAD	CGCTCGAGGAATTCGGTACCAGTACTGCTCGACGTTGGCCTT
zx-f	GGTAACATGATAGATCATGTCATTGTG
zx-r	TACAACGTGCACAACAGA
hyg501-f	GAGCATATACGCCCCGGAGTC
hyg501-r	CAAGACCTGCCTGAAACCGA
qCof-F	ACATCAACCGCGACAACTACGAG
qCof-R	TCCTTGTCGGCGTCCACGAA
qCAD5-F	CAACATCGAGACGCCGCCTGA
qCAD5-R	CACGAGCATGTACCATATCAAGT
1-F	TCTCGATATTCCACGCTGA
1-R	GCAACATGAACAATGCGTTT
2-F	GCGACCTCCTCACTAACCC
2-R	CTGATCACTCGCAGGTCCGTA
3-F	CTGCCGCATATATACATCC
3-R	AATTATTCATCTTTAAACGGAG
4-F	TGCCGGAGATCAAGTTCACCA
4-R	CCTTGTCTGCCTCTGCGATG
5-F	AGTTAATGAAGGCATGTAAACC
5-R	AGTACGGAAAGTAATTATCACA
6-F	GAGCGCCAACCCATTTAACAC
6-R	AGACAGCTCCATGGCTACAGA
7-F	GAGCGCCAACCCATTTAACAC
7-R	AGACAGCTCCATGGCTACAGA
CCoAOMT2-GFP-F	GGGGTACCATGGCCACCGCGACAG
CCoAOMT2-GFP-R	GCTCTAGAGGCAGATCTCGACGCGCTCA
CAD5-GFP-F	GGGGTACCATGGGCAGCGTCGCTTCGGAGA
CAD5-GFP-R	GCTCTAGAGCTAAGCCAGGTCGCTAGCCGTGGA
yz-F	ATCATCCTCGGCATGCAC
yz-R	CTCTGCAGGTCGACACT

It represents a sequence complementary to forward insertion fragment-F. It represents a sequence complementary to forward insertion fragment-R. It represents a sequence complementary to cof reverse insertion fragment-F. It represents a sequence complementary to cof reverse insertion fragment-R.

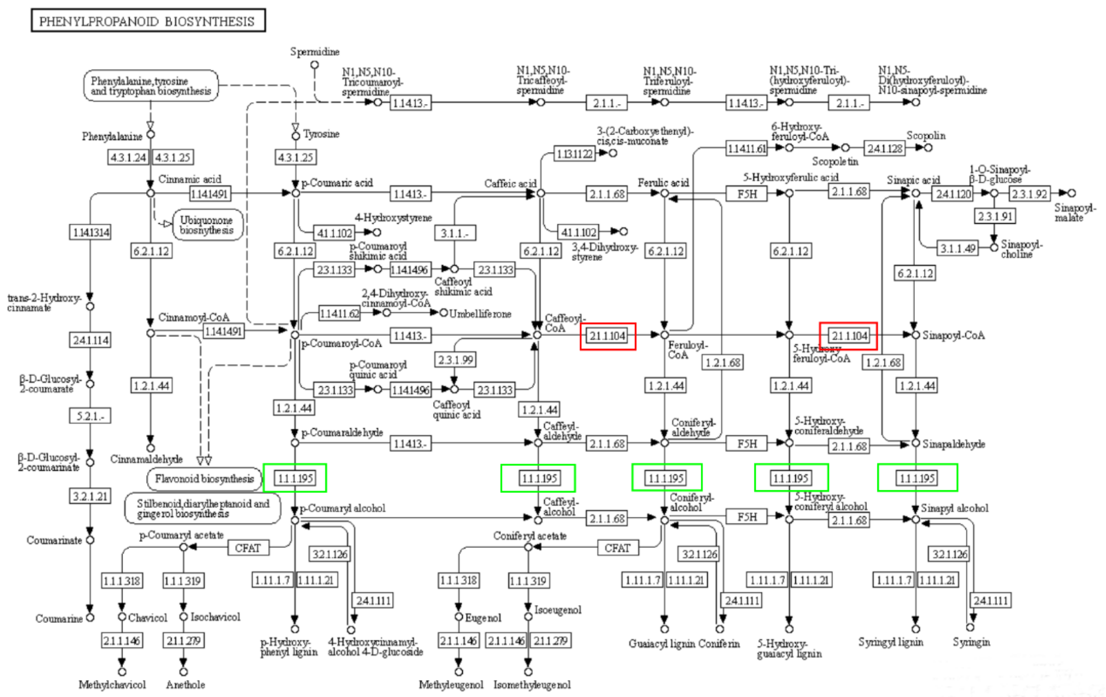

Figure S1. Phenylpropanoid Biosynthesis KEGG pass way.

The red box indicates the location of CCoAOMT2 in the pass way, the green box indicates the location of CAD5 in the pass way

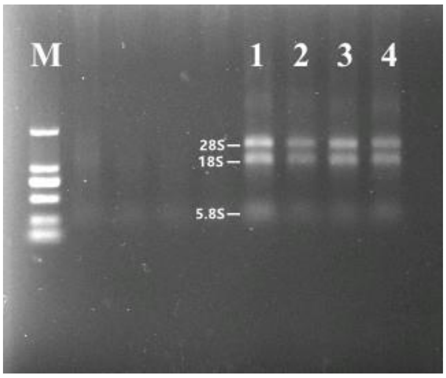

Figure S2. Electrophoresis diagram of RNA extraction from stem tip of *B. pervariabilis* ×*D. grandis*.

M: DL2000 DNA marker; 1, 2, 3 and 4: *B. pervariabilis* ×*D. grandis* RNA.

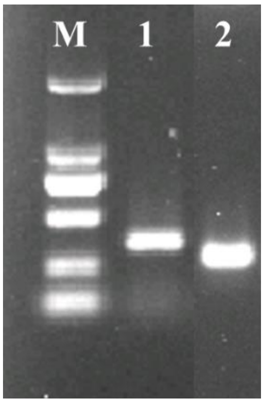

Figure S3. Electrophoretic image of amplified product of target fragment.

M: DL2000 DNA marker; 1: *CCoAOMT2* interferes with the target fragment; 2: *CAD5* interferes with the target fragment.

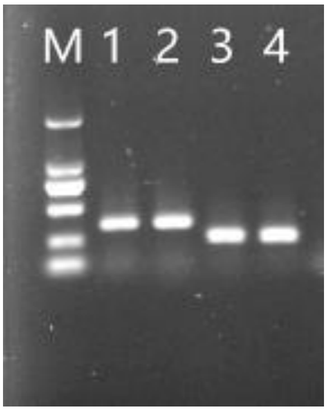

Figure S4. Electrophoretic images of cof and cad forward and reverse fragment amplification products.

M: DL2000 DNA marker; 1, 2, 3 and 4 are the forward and reverse insertion fragments of cof intermediate carrier and the forward and reverse insertion fragments of cad intermediate carrier, respectively.

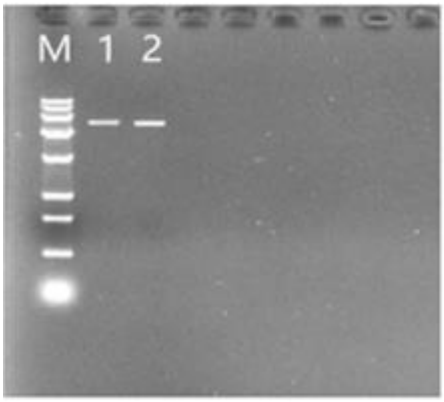

Figure S5. Linearized hinnabal-r-cof, hinnabal-r-cad vector.

M: DL 15000 DNA marker; 1: The recombinant plasmid of hinnabal-f-cof was digested by XbaⅠ; 2: The recombinant plasmid of phinnabal-f-cad was purified by XbaⅠ single enzyme.1

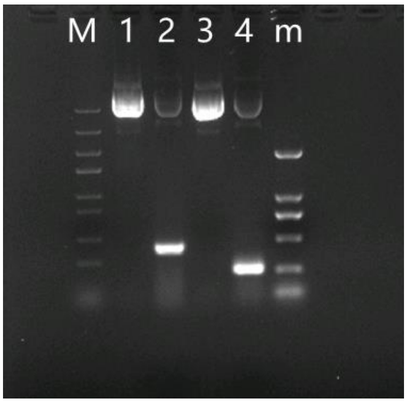

Figure S6. pHINNABAL-R-cof, pHINNABAL-R-cad vector and PCR detection results of the vector.

M: DL 15000 DNA marker; m: DL2000 DNA marker; 1: recombinant vector phinnabal-r-cof; 2: PCR results of recombinant vector phinnabal-r-cof plasmid; 3: Recombinant vector phinnabal-r-cad; 4: PCR results of phinnabal-r-cad plasmid.

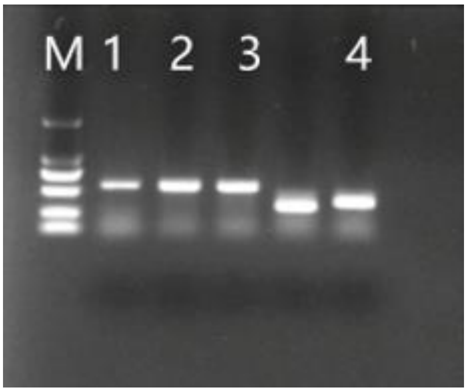

Figure S7. Forward insert PCR validation.

M: DL2000 DNA marker; 1, 2, 3: PCR results of recombinant intermediate vector phinnabal-f / r-cof plasmid; 4: PCR results of recombinant intermediate vector phinnabal-f / r-cad plasmid.

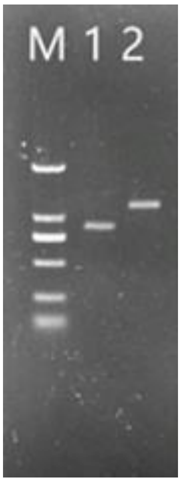

Figure S8. PCR amplification of CCOAOMT2 gene and CAD5 gene.

M: DL2000 DNA marker; 1: Ccoaomt2 complete CDS area; 2: CAD5 complete CDS area.
